# Insufficiency Fracture in the Para-Acetabulum, with Features Mimicking Those of a Malignant Bone Tumor

**DOI:** 10.4137/ccrep.s803

**Published:** 2008-06-06

**Authors:** Akio Sakamoto, Takuaki Yamamoto, Kazuhiro Tanaka, Shuichi Matsuda, Tatsuya Yoshida, Yukihide Iwamoto

**Affiliations:** Department of Orthopaedic Surgery, Graduate School of Medical Sciences, Kyushu University, Fukuoka, Japan.

**Keywords:** insufficiency fracture, para-acetabulum, plain radiograph, CT, MRI

## Abstract

Para-acetabular insufficiency fractures are rare and exceedingly difficult to diagnose without a high index of suspicion, since the images mimic those of bone tumors. We herein present the case of a 55-year-old woman who suffered from hip pain with subacute onset. She had undergone a hysterectomy-ovariectomy due to endometriosis when she was 41 years old. Her bone mineral density was normal due to supplemental treatment with female hormones. About 3 months after onset, she was referred to our institute with a diagnosis of pelvic bone tumor. Plain radiographs and computed tomography showed irregular osteosclerosis in the para-acetabulum. Bone scintigraphy demonstrated uptake in the para-acetabulum. Magnetic resonance imaging showed abnormal signal with low-signal intensity on T1-weighted images and high-signal intensity on T2-weighted images throughout the entire hemipelvic bone. Since the pain continued for more than 3 months, open biopsy was undertaken and the lesion was found to be non-neoplastic. Six months after onset, the pain disappeared. The clinical course suggested a diagnosis of insufficiency fracture in the para-acetabulum. Para-acetabular insufficiency fractures should always be considered in cases of hip pain, even in patients with prolonged symptoms.

## Introduction

Insufficiency fractures occur when normal stress is placed on a bone with deficient elastic resistance (Daffner RH and Pavlov H, 1992). Insufficiency fractures are most often seen in elderly women with postmenopausal osteoporosis. Underlying conditions associated with weakened bones are rheumatoid arthritis, radiation therapy and corticosteroid medications (Schreiber S, 1992). Para-acetabular insufficiency fractures are also reported to be commonplace among endurance trainees in military populations (Williams TR et al. 2002).

Pelvic insufficiency fractures most commonly occur in the sacrum (Mammone JF and Schweitzer ME, 1995), ilium and pubic bones (Fu AL et al. 1994). Although weight-bearing in the pelvis results in transmission of most of the mechanical load to the acetabulum (Mumber MP et al. 1997), para-acetabular insufficiency fractures are not common, especially as isolated insufficiency fractures. Para-acetabular insufficiency fractures are particularly difficult to diagnose, if they are not actually suspected, since the plain radiographic features are not specific. We herein report a case of insufficiency fracture of the para-acetabulum. The diagnosis was not made until follow-up, after open biopsy had been performed, since the features of this lesion mimic those of a bone tumor.

## Case Report

**Figure 1:**
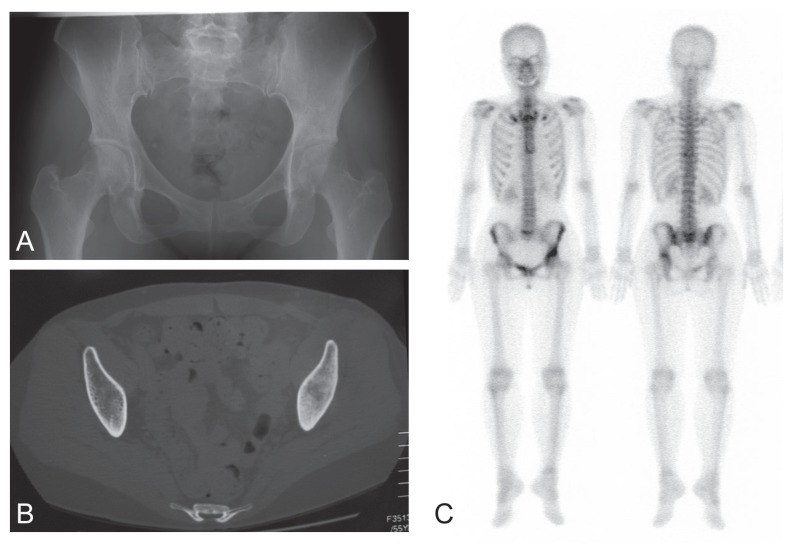
Plain radiographs show ill-defined sclerosis in the left para-acetabular region (**A**). CT shows diffuse irregular osteosclerosis in the left para-acetabular region, possibly associated with the fracture (**B**). Bone scintigraphy shows a markedly increased uptake in the left acetabulum (**C**).

**Figure 2:**
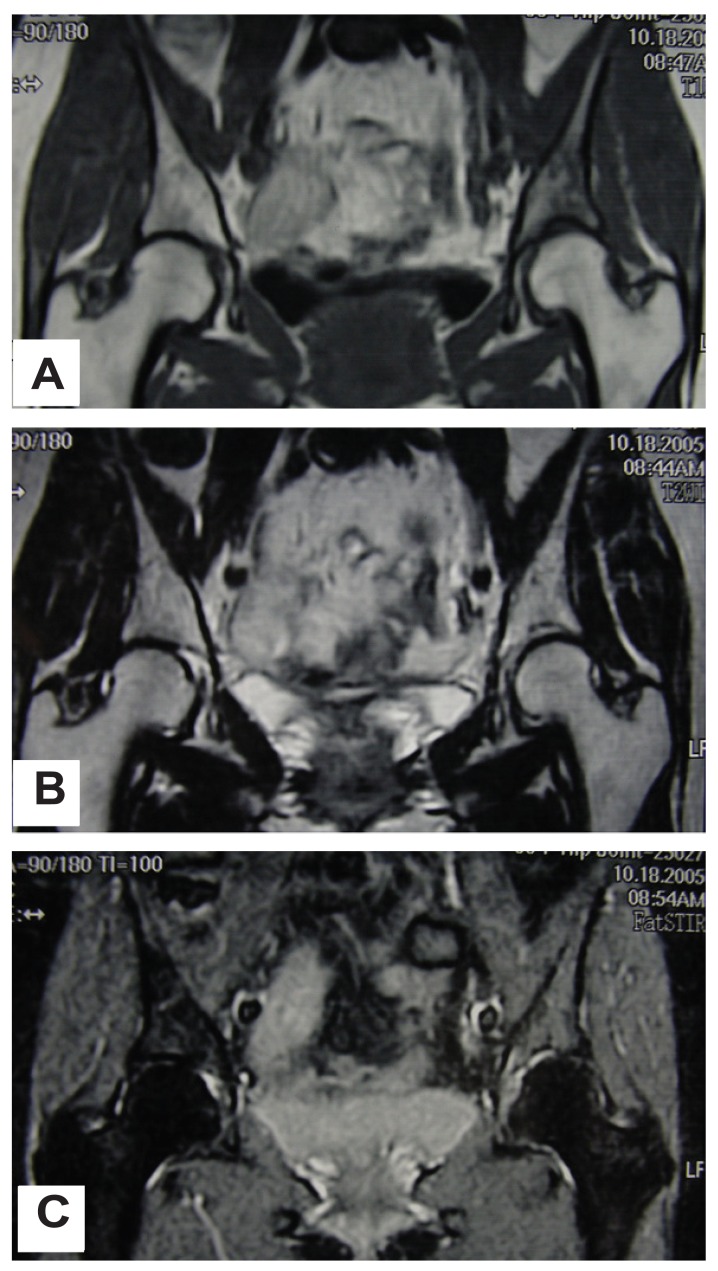
MRI shows diffuse low-signal intensity on T1-weighted image (**A**) and high-signal intensity on T2-weighted (**B**) and STIR images (**C**), suggesting bone marrow edema, associated with the fracture.

We present the case of a 55-year-old woman who suffered from hip pain with subacute onset. She was 150 cm tall and weighed 46.0 kg (body mass index = 20.4). She had undergone hysterectomy-ovariectomy due to endometriosis when she was 41 years old. Female hormones had been administered ever since. Her bone mineral density was normal at the trochantric region of the femur (bone mineral density, 0.776 g/cm^2^; T-score, −0.8; Z-score, 0.3; age-matched, 104%). The patient did not take any calcium supplements nor osteoporosis agents, such as alendronate or calcitonin. There was no family history of bone disease. About 3 months after onset, she was referred to our institute with the diagnosis of a bone tumor. Plain radiographs showed irregular osteosclerosis in the para-acetabulum (Fig. 1A). Computed tomography (CT) also showed diffuse osteosclerosis in the para-acetabulum (Fig. 1B). Bone scintigraphy demonstrated uptake in the para-acetabulum (Fig. 1C). Magnetic resonance imaging (MRI) showed a low-signal intensity on T1-weighted images and a high-signal intensity on T2-weighted and STIR images throughout the entire hemipelvic bone (Fig. 2).

Serum tumor markers of AFP, CA19-9 and CA125 were all within the normal limits. Because the pain continued for more than 3 months after onset, open biopsy was performed to rule out a neoplastic lesion. Biopsy findings showed that the lesion was actually a non-neoplastic lesion. The patient was treated by avoidance of weight-bearing, and the pain disappeared 6 months after onset. MRI 8 months after onset demonstrated a signal intensity that was almost normal (data not shown). The clinical course suggested that the correct diagnosis was insufficiency fracture in the para-acetabulum. Consent for the publication of the data of this case was obtained from the patient.

## Discussion

Insufficiency fractures are most often seen in elderly women with postmenopausal osteoporosis. Although the current case had undergone hysterectomy-ovariectomy, her bone mineral density was normal because she had been receiving female hormone supplements. However, abnormality of bone mineral density is defined based upon age-matched data. Therefore, it is true that she demonstrated some osteopenia considering that she is a 55-year-old postmenopausal woman.

Hip pain is a common diagnostic challenge that may be related to many different disorders affecting the joint itself, periarticular soft tissues or even distant anatomic structures (Theodorou SJ et al. 2006). Plain radiograph is relatively insensitive to stress fractures at an early stage, especially in the pelvis which is a complex anatomical structure (Anderson MW and Greenspan A, 1996), and the lesion can easily be overlooked (Williams TR et al. 2002; Otte MT et al. 1997; Theodorou SJ et al. 2006). Moreover, plain radiographs are inadequate for distinguishing between early insufficiency fractures and metastatic bone tumors (Theodorou SJ et al. 2006; Kiuru MJ et al. 2002; Kiuru MJ et al. 2003). Bone scintigraphy is a sensitive approach with which to identify the location of an insufficiency fracture, but its specificity is low (Anderson MW and Greenspan A, 1996). In the later stages of an insufficiency fracture in which osteosclerosis develops, the osteosclerosis seen on a plain radiograph may be diagnosed as osteoblastic metastasis or a primary bone tumor (Fu AL et al. 1994). CT is used to delineate a fracture line in the complex anatomical region of the acetabulum (Theodorou SJ et al. 2006). MRI is also a useful imaging technique in the diagnosis of insufficiency fractures in the pelvis (Williams TR et al. 2002; Otte MT et al. 1997; Kiuru MJ et al. 2002; Kiuru MJ et al. 2003). MRI examination is recommended in all 3 basic planes (coronary, axial and sagittal) including Gadolinium-enhanced T1-weighted imaging, with and without fat suppression, in the pelvis with complex anatomical structure. Typically, identification of a linear lesion with low-signal intensity on both T1- and T2-weighted images is characteristic of a fracture line in an insufficiency fracture. Moreover, a diffuse area with low-signal intensity on T1-weighted images and high-signal intensity on T2-weighted images, suggesting bone marrow edema, is usually seen surrounding the fracture line (Otte MT et al. 1997).

In the current case, plain radiograph and CT showed irregular ostesclerosis in the para-acetabulum, but failed to demonstrate a clear fracture line. Bone scintigraphy demonstrated uptake in the lesion. MRI showed only a diffuse lesion with low-signal intensity on T1-weighted images and high-signal intensity on T2-weighted images, without demonstrating a fracture line. Without the fracture line of low-signal intensity on T1- and T2-weighted images, the features of MRI are not distinguishable between those of insufficiency fractures and those of bone tumors. It has been reported that the symptoms of insufficiency fracture resolve within approximately 3 months (Otte MT et al. 1997). However, the pain in the current case continued for more than 3 months. This led us to performed open biopsy for the purpose of diagnosis, and we were thus able to rule out a neoplastic lesion, such as a metastatic bone tumor or a lymphoma. As an alternative to open biopsy, CT-guided transcutaneous biopsy would be another choice in such cases. When making a histological diagnosis, one should bear in mind that the results of a biopsy of a healing fracture can be misleading and can suggest osteosarcoma or chondrosarcoma, since the histological features of these tumors look quite similar (Theodorou SJ et al. 2006).

We present the case of a 55-year-old woman with para-acetabular insufficiency fracture. The prolonged symptoms and diffuse abnormal images of MRI may lead to misdiagnosis as a malignant tumor. In addition, plain radiographs of insufficiency fractures generally mimic those of malignant bone tumors at any stage. Maintaining a high degree of clinical suspicion is required in order to make a correct diagnosis of an insufficiency fracture, even in patients who have prolonged clinical symptoms.
